# A nationwide survey on the management of neonatal respiratory distress syndrome: insights from the MUNICH survey in 394 Chinese hospitals

**DOI:** 10.1186/s13052-024-01741-7

**Published:** 2024-09-07

**Authors:** Long Chen, Yong Ji, Rong Ju, Jiang-Qin Liu, Ling Liu, Jingyun Shi, Hui Wu, Lili Wang, Falin Xu, Chuanzhong Yang, Huayan Zhang, Yuan Shi, MUNICH Study Group

**Affiliations:** 1 Department of Neonatology, Women and Children’s Hospital of Chongqing Medical University, Chongqing Health Center for Women and Children, No.120 Longshan Road, Yubei District, Chongqing, China; 2 Department of Neonatology, Children’s Hospital of Shanxi, No.310 Changzhi Road, Xiaodian District, Taiyuan, Shanxi China; 3 Department of Neonatology, Chengdu Women’s and Children’s Central Hospital, No.1617 Riyue Avenue, Qingyang District, Chengdu, Sichuan China; 4 Department of Neonatology, Shanghai First Maternity and Infant Hospital, No.2699 West Gaoke Road, Pudong District, Shanghai, China; 5 Department of Neonatology, Guiyang Maternal and Child Health Care Hospital-Guiyang Children’s Hospital, No.63 South Ruijin Road, Nanming District, Guiyang, Guizhou China; 6 Department of Neonatology, Gansu Provincial Maternal and Child Care Hospital (Gansu Provincial Central Hospital), No.143 North Qilihe Street, Lanzhou, Gansu, China; 7 Department of Neonatology, The First Hospital of Jilin University, No.1 Xinmin Street, Changchun, Jilin, China; 8 Department of Neonatology, The First Affiliated Hospital of Anhui Medical University, No.218 Jixi Road, Shushan District, Hefei, Anhui China; 9 Department of Neonatology, The Third Affiliated Hospital of Zhengzhou University, No.7, Kangfuqian Street, Erqi District, Zhengzhou, Henan China; 10 Department of Neonatology, Shenzhen Maternity & Child Healthcare Hospital, No.2004 Hongli Road, Futian District, Shenzhen, Guangdong China; 11 Department of Neonatology, Guangzhou Women and Children’s Medical Center, National Children’s Medical Center for South Central Region, No.9 Jinsui Road, Zhujiang New Town, Guangzhou, Guangdong China; 12 Division of Neonatology, Children’s Hospital of Philadelphia, 3401 Civic Center Blvd, Philadelphia, USA; 13 Department of Neonatology, Children’s Hospital of Chongqing Medical University, National Clinical Research Center for Child Health and Disorders, Ministry of Education Key Laboratory of Child Development and Disorders, No.136 Zhongshan Second Road, Yuzhong District, Chongqing, China

**Keywords:** Respiratory distress syndrome, Neonate, Surfactant, Ventilation, Epidemiology

## Abstract

**Background:**

At present, preterm infants with respiratory distress syndrome (RDS) in China present higher mortality and morbidity rates than those in high-income countries. The aim of this nationwide survey was to assess the clinical management of RDS in China.

**Methods:**

A nationwide cross-sectional survey to assess adherence to RDS management recommendations was performed. One neonatologist per hospital was randomly selected. The primary outcome was the key care of RDS management.

**Results:**

Among the 394 participating hospitals, 88·3% were birthing centres. The number of doctors and nurses per bed were 0·27 and 0·72, respectively. Antenatal corticosteroids (any dose) were administered to 90% of the women at risk of preterm birth at < 34 weeks of gestation (90·0% inborn vs. 50·0% outborn, *p* < 0·001). The median fraction of inspired oxygen (FiO_2_) for initial resuscitation was 0·30 for babies born at ≤ 32 weeks of gestation and 0·25 for those born at > 32 weeks. T-piece resuscitators were available in 77·8% of delivery rooms (DRs) (tertiary hospitals: 82·5% vs. secondary hospitals: 63·0%, *p* < 0·001). Surfactant was used in 51·6% of the DRs. Less invasive surfactant administration (LISA) was used in 49·7% of the hospitals (tertiary hospitals: 55·3% vs. secondary hospitals: 31·5%, *p* < 0·001). Primary non-invasive ventilation was initiated in approximately 80·0% of the patients. High-frequency oscillation ventilation was primarily reserved for rescue after conventional mechanical ventilation (MV) failure. Caffeine was routinely used during MV in 59·1% of the hospitals. Bedside lung ultrasonography was performed in 54·3% of the health facilities (tertiary hospitals: 61·6% vs. secondary hospitals: 30·4%, *p* < 0·001). Qualified breast milk banks and Family Integrated Care (FICare) were present in 30·2% and 63·7% of the hospitals, respectively.

**Conclusions:**

Significant disparities in resource availability and guidelines adherence were evident across hospitals. Future strategies should address DR facilities and medication access, technical training, staff allocation, and ancillary facility development for a better management of RDS patients in China.

**Supplementary Information:**

The online version contains supplementary material available at 10.1186/s13052-024-01741-7.

## Background

Neonatal respiratory distress syndrome (RDS) is a prevalent pulmonary condition observed in preterm infants, and is primarily linked to insufficient pulmonary surfactant (PS) or underdeveloped lung structures [1, 2]. RDS affects approximately 30% of infants born between 28 and 34 weeks of gestation, with the prevalence increasing to approximately 60% for those born before 28 weeks [3]. The Chinese Neonatal Network (CHNN) reported a survival rate of 87·6%, with 51·8% of infants born at < 32 weeks of gestation surviving without major morbidities [4]. However, survival rates vary with socioeconomic status. In low- and middle-income countries (LMICs), more than 90% of extremely preterm infants (EPIs, less than 28 weeks of gestation) do not survive beyond the first few days of life, with RDS being one of the most common causes of death, compared with the mortality rate of less than 10% in high-income countries [5]. Even in high-income countries, critical conditions associated with significant morbidity and mortality still exist in specific high-risk perinatal situations (e.g., specific social or geographic reasons) [6]. Efforts to standardize RDS management, including prenatal care, stabilization in the delivery room (DR), surfactant administration, and ventilation strategies, have led to the establishment of various guidelines and consensuses [7–9].

Perinatal and neonatal care in China has significantly progressed, with neonatal mortality rate of 3·1‰ in 2021, closely aligning with the rates in high-income countries [10, 11]. Local guidelines and consensuses in China contribute to defining the criteria for RDS care and neonatal intensive care unit (NICU) construction [7, 12]. National and regional neonatal networks are actively engaged in fostering quality improvements in participating hospitals [4, 12–14]. Additionally, the development of regional neonatal transportation systems development reflects improved management for critically ill neonates.

Nevertheless, numerous lower-tier hospitals in China continue to face challenges in providing timely and effective treatment, particularly for EPIs [15]. Given the vast size and population of China, the Medical sUrvey of NICU Insight in CHina (MUNICH) was conducted to comprehensively explore current RDS management across the country.

## Methods

### Study design and participants

In China, general hospitals and maternity-child healthcare hospitals are birthing centres where pregnant women give birth. In contrast, all neonates in children’s hospitals are outborn and transferred from birthing centres not only due to respiratory diseases, but also due to other congenital defects or perinatal diseases [16–18]. Maternity-child healthcare hospitals and children’s hospitals are usually referred to as paediatric specialty hospitals, which provide treatment for children as well as neonates. Physicians may work as specialists in neonatology departments or as paediatricians responsible for both neonates and children.

In China, tertiary hospitals provide high-level specialized medical services to several areas where most high-risk pregnant women and preterm babies are treated. In parallel, secondary hospitals are regional hospitals that provide comprehensive health services to multiple communities. Although most preterm babies born in secondary hospitals are transferred to designated tertiary hospitals, physicians in secondary hospitals may occasionally deal with preterm birth. Cities were categorized into 1st-tier, 2nd-tier, and 3rd-tier and lower-tier cities. 1st-tier and 2nd-tier cities were defined as well-developed cities, including municipalities directly under the Central Government, capital cities, or regional centres (Table S1). Mainland China was stratified according to a traditional seven-region partition (Northeast, North, East, South, Central, Northwest, and Southwest China). The provinces/autonomous regions/centrally administered municipalities in each region are listed in Table S2. Doctors’ titles were divided into chief and associate chief physician and attending and resident physicians.

On the basis of the above background, a nationwide, multicentre, cross-sectional survey was designed. We used the online MedSci database, covering 31 provincial administrative regions, municipalities, and autonomous regions in China, with 95,444 registered doctors. A total of 2,881 hospitals with neonatal units were identified in the MedSci database.

The MUNICH was conducted with stratified convenience sampling according to the different regions and types of hospitals. The sample size was allocated to eligible hospitals in each region, and only one doctor was recruited at each hospital. The adjusted Yamane formula in the equation was used for sample size calculation.

$$\text{n }=\frac{N}{1+N{\varepsilon }^{2}}$$

where

n = sample size

N = population size = 1500

e = the degree of accuracy expressed as a proportion = 0.03

$\rho$ = the number of standard deviations that would include all possible values in the range of 4

t = t-value for the selected alpha level or confidence level at 99% = 2.58

ɛ = adjust margin of error [(ε = $\frac{\rho e}{t}$)]

**Fig. 1:**
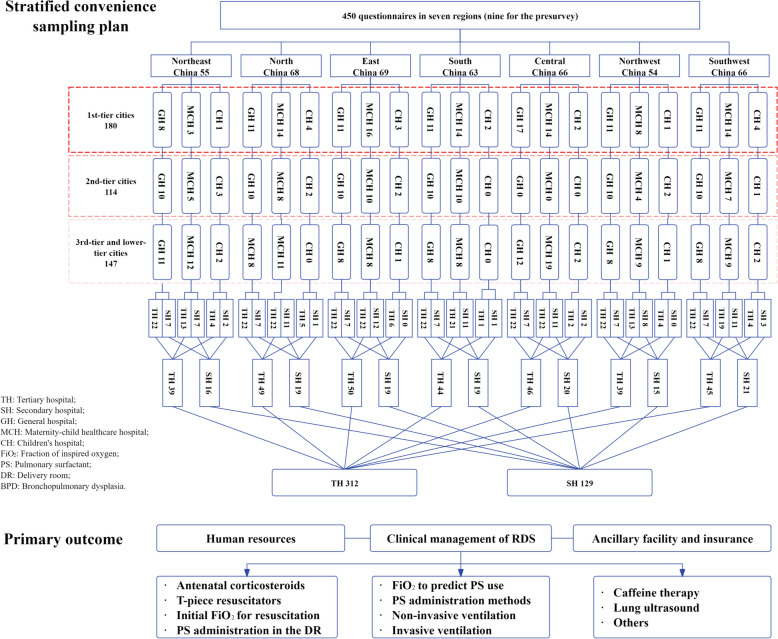
Sampling plan and primary outcome of the MUNICH

The estimated effective sample size of 354 participants was required to achieve a 99% confidence interval (CI), assuming a target hospital number of 1500 (200–250 hospitals in each of the seven district regions). The estimated questionnaire response rate was 90%, and 90% of the responses was valid, for a total sample size of 437. Considering the diverse development across various regions in China and the variability in hospital types, a total sample size of 450 cases was selected in this study. 50–70 doctors from public hospitals in each region were planned to be recruited, with a total of 450 doctors in seven regions (nine doctors for the presurvey). The sample was divided into general hospitals, maternity-child healthcare hospitals and children’s hospitals at a ratio of 6:6:1, and into tertiary and secondary hospitals at a ratio of 3:1 in each region (Fig. 1). Physicians who worked in the neonatology department of the selected hospitals were randomly chosen. If the first doctor did not respond, a second doctor from the same hospital was contacted. If no doctor in the selected hospital responded, a doctor at a backup hospital was contacted. Data collection spanned from October to December 2022.

In line with the principles of the Declaration of Helsinki, the study was approved by the Ethics Committee of the Children’s Hospital of Chongqing Medical University (Approval Number: 2022–376). All the physicians provided informed consent before participating in the survey.

### Questionnaire preparation

The questionnaire was collaboratively developed by the MUNICH Study Group, which comprised specialists with extensive expertise in RDS management. The questionnaire, in alignment with established guidelines, underwent rigorous assessment. A preliminary survey involving nine neonatologists was performed to ensure data clarity and precision. The questionnaire was composed of six dimensions: general information (11 questions), prenatal and perinatal conditions (20 questions), non-invasive ventilation (eight questions), invasive ventilation (eight questions), pulmonary surfactant administration (21 questions), and others (15 questions) (Supplemental 1).

### Questionnaire distribution and data collection

A third-party online survey platform was used to manage the distribution and collection of questionnaires. Doctors received the questionnaire via a web link or QR code. The exclusion criteria were as follows: (1) survey with the same answer option selected for all questions or answer options selected with apparent regularity (e.g., ABAB or AAAA); (2) surveys for which the respondent failed to follow the instructions for the question or answered outside the scope of the question; and (3) survey responses from doctors at private hospitals and nurses.

### Statistical analyses

Categorical data were represented as frequencies (N) and percentages (%), and intergroup differences were assessed via the chi-square test. Continuous data normality was determined via the Shapiro‒Wilk method. Normally distributed continuous data were expressed as the mean ± standard deviation (mean ± SDs) and were analyzed via t tests or analysis of variance. Nonnormally distributed continuous data were presented as the median (median), first quartile (Q1), and third quartile (Q3), with intergroup comparisons conducted via the Mann‒Whitney U test or Kruskal‒Wallis H test. All the statistical tests were two-tailed, adhering to a predetermined significance level of α = 0·05. The average composite score for the ranking questions was calculated as follows: composite score = (Σ frequency × weight)/number of people who answered the question. The frequency was the number of people who chose each option to be ranked in different positions. The weight was determined by the ranking of the options. For example, if there were three options involved, the weight of the first position in the ranking was three, the second was two, and the third was one. Statistical analysis and boxplots were performed via R (Version 4·2·2). All the data were analysed in five dimensions: hospital level, hospital type, city tier, geographical region, and doctor title.

## Results

### Profiles of the included physicians and hospitals

Of the 449 questionnaires distributed, 398 responses were obtained (nine presurvey questionnaires were not included). Four questionnaires were excluded (two from nurses and two from doctors at private hospitals), resulting in 394 valid responses from 30 provinces across China (Table S1). The response rate was 88·9% (407/458), and 99·0% of the responses were valid (394/398). Among the respondents, 378 were from birthing centres and 16 were from children’s hospitals. Among all the physicians, 97·4% (384/394) were from the neonatology department or neonatal intensive care unit. Tertiary hospitals constituted 60·9% (298/394) of the sample. Further details on the participating doctors and hospitals are provided in Table 1.

**Table 1:** Characteristics of the physicians and hospitals included in the survey

	**Population (*N* = 394)**
**Sex, n (%)**	
Male	151 (38·3)
Female	243 (61·7)
**Age (years)**	
** Median (Q1, Q3)**	**43·00 (39·00, 50·00)**
**Hospital type, n (%)**	
Maternity-child healthcare hospital	137 (34·8)
Children’s hospital	16 (4.1)
General hospital	241 (61·2)
**Hospital level, n (%)**	
Tertiary hospital	240 (60·9)
Secondary hospital	154 (39·0)
**City tier, n (%)**	
1st	131 (33·2)
2nd	55 (14·0)
3rd and lower-tier	208 (52·8)
**Geographical region**	
Northeast China	39 (9·9)
North China	49 (12·4)
East China	72 (18·3)
South China	53 (13·5)
Central China	55 (14·0)
Northwest China	53 (13·5)
Southwest China	73 (18·5)
**Doctor’ title**	
Chief and associate chief physician	273 (69·3)
Attending and resident physician	121 (30·7)
**Department, n (%)**	
Neonatology	372 (94·4)
NICU	12 (3·0)
Other	10 (2·5)

*NICU* Neonatal intensive care unit

### Bed capacity and human resources

The median numbers of NICUs and total beds were 10·0 (5·0, 25·0) and 30·0 (20·0, 50·0), respectively (Table 2). Compared with secondary hospitals, tertiary hospitals had more beds (20·0 (12·0, 25·0) vs. 35·0 (22·3, 60·0) beds, *p* < 0·001). There were more beds in paediatric specialty hospitals than in general hospitals (40·0 (25·0, 80·0) vs. 25·0 (17·0, 40·0) beds, *p* < 0·001).

**Table 2:** Comparison of bed capacity and human resources

	***n* = 394**	**Hospital tier**			**Hospital type**			**City tier**			
		**Tertiary hospital**	**Secondary hospital**	***P***	**General hospital**	**Paediatric specialty hospital**	***P***	**1st-tier cities**	**2nd-tier cities**	**3rd-tier and lower-tier cities**	***P***
**Numbers**											
Number of beds, Median (Q1, Q3)	30·0 (20·0, 50·0)	35·0 (22·3, 60·0)	20·0 (12·0, 25·0)	< 0·001	25·0 (17·0, 40·0)	40·0 (25·0, 80·0)	< 0·001	30·0 (18·0, 67·0)	32·0 (21·5, 50·0)	30·0 (20·0, 45·0)	0·069
Number of doctors, Median (Q1, Q3)	8·0 (6·0, 13·0)	10·0 (7·0, 16·0)	5·0 (4·0, 7·0)	< 0·001	8·0 (5·0, 10·0)	10·0 (6·0, 19·0)	< 0·001	9·0 (6·0, 19·5)	10·0 (6·0, 16·0)	8·0 (5·8, 10·25)	0·002
Number of nurses, Median (Q1, Q3)	20·0 (14·0, 36·0)	25·0 (16·3, 42·0)	12·5 (8·0, 18·0)	< 0·001	19·0 (12·0, 30·0)	25·0 (16·0, 48·0)	< 0·001	20·0 (14·0, 54·0)	26·0 (14·0, 39·5)	20·0 (13·0, 30·0)	0·155
**Ratio**											
Doctors per bed, Median (Q1, Q3)	0·27 (0·20, 0·37)	0·27 (0·20, 0·37)	0·30 (0·22, 0·40)	0·255	0·29 (0·22, 0·40)	0·26 (0·20, 0·33)	0·002	0·30 (0·22, 0·40)	0·25 (0·20, 0·33)	0·27 (0·21, 0·35)	0·336
Nurses per bed, Median (Q1, Q3)	0·72 (0·56, 0·90)	0·73 (0·58, 0·90)	0·67 (0·53, 0·85)	0·139	0·77 (0·60, 0·93)	0·66 (0·53, 0·80)	< 0·001	0·71 (0·53, 0·89)	0·69 (0·52, 0·94)	0·72 (0·60, 0·90)	0·472

**Fig. 2:**
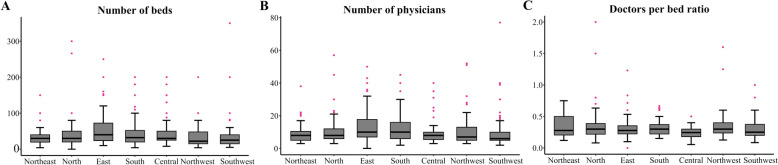
Variations in human resources among the different geographic regions (*p* < 0·05). **a** Number of beds, **b** Number of doctors, **c** Doctors per bed ratio

The median numbers of doctors per bed and nurses per bed were 0·27 and 0·72, respectively. Paediatric specialty hospitals had higher ratios than general hospitals did (doctors per bed, 0·26 vs. 0·29, *p* = 0·002; nurses per bed, 0·66 vs. 0·77, *p* < 0·001). Comparisons of bed capacity and human resources among different regions are shown in Fig. 2 and Table S3.

### Main points of RDS care

#### Antenatal corticosteroids

Antenatal corticosteroids (any dose) were administered to approximately 90% of the women at risk of preterm birth at < 34 weeks of gestation. Among the 176 hospitals with both outborn and inborn babies, fewer outborn babies than inborn babies received antenatal corticosteroids (50·0% vs. 95·0%, *p* < 0·001). The reasons for the lack of antenatal corticosteroid use are shown in Fig. S1A, with precipitous labour exhibiting the highest score of 4·64 points.

#### Delivery rooms

Data related to DRs were obtained from 378 birthing centres.

##### Oxygen therapy in DRs

Oxygen blenders were present in 82·0% (310/378) of the DRs in the birthing centres. A median fraction of inspired oxygen (FiO_2_) of 0·30 for babies born at < 28 weeks of gestation, 0·30 for those born at 28–31 weeks of gestation, and 0·25 for those born at > 32 weeks of gestation was used for initial resuscitation. Notably, general hospitals preferred to set higher initial FiO_2_ values than maternity-child healthcare hospitals did for preterm babies born between 28–31 weeks of gestation (0·30 (0·30, 0·40) vs. 0·30 (0·25, 0·35), *p* = 0·023) and > 32 weeks of gestation (0·30 (0·21, 0·40) vs. 0·21 (0·21, 0·30), *p* = 0·001). The designated lower and upper limits for target oxygen saturation after the first 10 min postnatally were 89% and 95%, respectively. Compared with secondary hospitals, tertiary hospitals exhibited higher limits (88·0%-95·0% vs. 90·0%-95·0%, *p* < 0·05). The initial FiO_2_ and target oxygen saturation limits among the different groups of hospitals are shown in Table 3.

**Table 3:** Comparison of resuscitation strategies in the delivery room

		**Hospital tier**			**Hospital type**			**City tier**			
		**Tertiary hospital**	**Secondary hospital**	***P***	**General hospital**	**Paediatric specialty hospital**	***P***	**1st-tier cities**	**2nd-tier cities**	**3rd-tier and lower-tier cities**	***P***
**Oxygen therapy in the DR**											
* Initial FiO*_*2*_* for resuscitation*^b^* (n* = *310)*	*n* = 310	*n* = 237	*n* = 73		*n* = 222	*n* = 88		*n* = 101	*n* = 43	*n* = 166	
GA < 28w, Median (Q1, Q3)	0·30 (0·30, 0·40)	0·30 (0·30, 0·40)	0·31 (0·30, 0·40)	0·28	0·30 (0·30, 0·40)	0·30 (0·30, 0·40)	0·506	0·30 (0·30, 0·40)	0·30 (0·30, 0·40)	0·30 (0·30, 0·40)	0·547
GA 28-32w, Median (Q1, Q3)	0·30 (0·30, 0·40)	0·30 (0·30, 0·38)	0·30 (0·28, 0·40)	0·132	0·30 (0·30, 0·40)	0·30 (0·25, 0·35)	0·023	0·30 (0·25, 0·40)	0·30 (0·25, 0·35)	0·30 (0·30, 0·40)	0·340
GA > 32w, Median (Q1, Q3)	0·25 (0·21, 0·37)	0·25 (0·21, 0·30)	0·30 (0·21, 0·40)	0·405	0·30 (0·21, 0·40)	0·21 (0·21, 0·30)	0·001	0·22 (0·21, 0·30)	0·25 (0·21, 0·30)	0·30 (0·21, 0·40)	0·191
* Target saturation*^a^	*n* = 378	*n* = 286	*n* = 92		*n* = 241	*n* = 137		*n* = 131	*n* = 55	*n* = 208	
Lower limit, Median% (Q1, Q3)	89·0 (85·25, 90·0)	90·0 (87·0, 90·0)	88·0 (85·0, 90·0)	0·002	90·0 (86·0, 90·0)	89·0 (85·0, 90·0)	0·583	90·0 (87·5, 90·0)	90·0 (87·0, 90·0)	89·0 (85·0, 90·0)	0·167
Upper limit, Median% (Q1, Q3)	95·0 (94·0, 95·0)	95·0 (94·0, 95·0)	95·0 (93·0, 95·0)	0·016	95·0 (93·0, 95·0)	95·0 (94·0, 95·0)	0·31	95·0 (94·0, 95·0)	95·0 (94·0, 95·0)	95·0 (93·0, 95·0)	0·066
**Accessibility**^a^	*n* = 378	*n* = 286	*n* = 92		*n* = 241	*n* = 137		*n* = 131	*n* = 55	*n* = 208	
T-piece resuscitator	294/378 (77·8)	236/286 (82·5)	58/92 (63·0)	< 0·001	178/241 (73·9)	116/137 (84·7)	0·015	99/131 (83·2)	40/55 (78·4)	155/208 (74·5)	0·191
Surfactant in the DR	195/378 (51·6)	145/286 (50·7)	50/92 (54·3)	0·542	111/241 (46·1)	84/137 (61·3)	0·004	77/131 (64·7)	31/55 (60·8)	87/208 (41·8)	< 0·001

*DR* Delivery room, *FiO*_*2*_ Fraction of inspired oxygen, *GA* Gestational age

^a^378 Birthing centres

^b^310 Hospitals with an oxygen blender in the delivery room

##### T-piece resuscitators

T-piece resuscitators (TPRs) were available in 77·8% (294/378) of the DRs (tertiary hospitals: 82·5% vs. secondary hospitals: 63·0%, *p* < 0·001; general hospitals: 73·9% vs. maternity-child healthcare hospitals: 84·7%, *p* = 0·015). Forty-eight (48/378, 12·7%) respondents had only bag-valve-mask resuscitators for positive pressure ventilation in their DRs, with equipment deficiency being the most common cause (41/48, 85·4%). In Northwest China, access to TPRs was comparatively better, at a rate of 90·4%, which was significantly higher than the rates in other regions (*p* = 0·019) (Table S4).

##### PS administration in DRs

A total of 195 (51·6%) hospitals could use PS in the DR. Maternity-child healthcare hospitals had greater accessibility to PS than general hospitals did (61·3% vs. 46·1%, *p* = 0·004). Compared with 3rd tier and below cities, more hospitals in 1st-tier cities and 2nd-tier cities could use PS in the DR (41·8% vs. 64·7% vs. 60·8%, *p* < 0·001). The inability to obtain surfactant from the hospital pharmacy, lack of reimbursement before birth, and potential refusal to pay for medication (ranking scores: 4·15, 3·64, and 3·01, respectively) were the top three reasons for the absence of PS in the DRs (Fig. S1B).

#### PS administration

A total of 60·4% of the respondents used FiO_2_ (41·6% chose > 0·30, 41·2% > 0·40 and 15·5% > 0·50) as an indicator for PS administration in patients receiving non-invasive ventilation (NIV). PS was routinely administered to patients receiving mechanical ventilation (MV) according to 79·7% of the respondents.

As shown in Table 4, the INtubation-SURfactant-Extubation (INSURE) method was used in 341 (341/394, 86·5%) hospitals. Moreover, less invasive surfactant administration (LISA) and/or minimally invasive surfactant therapy (MIST) could be used in 49·7% (196/394) of the hospitals. Compared with secondary hospitals, tertiary hospitals presented a greater capacity for LISA/MIST (31·5% vs. 55·3%, *p* < 0·001). LISA/MIST was utilized in approximately 30·0% of RDS patients receiving NIV in hospitals performing LISA/MIST. Furthermore, feeding tubes were used for LISA/MIST in nearly half (50·5%) of the hospitals, whereas peripheral vein catheters and umbilical venous catheters were available in 28·2% and 19·8% of the hospitals, respectively.

**Table 4:** Accessibility and availability comparisons of techniques and ancillary facilities

	***n* = 394**	**Hospital tier**			**Hospital type**			**City tier**			
		**Tertiary hospital**	**Secondary hospital**	***p***	**General hospital**	**Paediatric specialty hospital**	***p***	**1st-tier cities**	**2nd-tier cities**	**3rd-tier and lower-tier cities**	***p***
INSURE	341/394 (86·5)	262/302 (86·8)	79/92 (85·9)	0·828	213/241 (88·4)	128/153 (83·7)	0·181	110/131(84·0)	47/55 (85·5)	184/208 (88·5)	0·482
LISA/MIST	196/394 (49·7)	167/302 (55·3)	29/92 (31·5)	< 0·001	108/241 (44·8)	88/153 (57·5)	0·014	69/131 (52·7)	30/55 (54·5)	97/208 (46·6)	0·415
LUS	214/394 (54·3)	186/302 (61·6)	28/92 (30·4)	< 0·001	124/241 (51·5)	90/153 (58·8)	0·152	73/131 (55·7)	35/55 (63·6)	106/208 (51·0)	0·226
Certified breast milk banks	119/394 (30·2)	101/302 (33·4)	18/92 (19·6)	0·011	63/241 (26·1)	56/153 (36·6)	0·028	48/131 (36·6)	19/55 (34·5)	52/208 (25·0)	0·057
Home-like wards	142/394 (36·0)	114/302 (37·7)	28/92 (30·4)	0·201	68/241 (28·2)	74/153 (48·4)	< 0·001	50/131 (38·2)	24/55 (43·6)	68/208 (32·7)	0·266
FICare	251/394 (63·7)	205/302 (67·9)	46/92 (50·0)	0·002	141/241 (58·5)	110/153 (71·9)	0·007	88/131 (67·2)	37/55 (67·3)	126/208 (60·6)	0·393

*DR* Delivery room, *INSURE* INtubation-SURfactant-Extubation, *LISA* Less invasive surfactant administration, *FiO*_*2*_ Fraction of inspired oxygen, *FICare* Family integrated care

#### Non-invasive ventilation

NIV was initiated as primary support in approximately 80·0% of the RDS patients (Central China had a higher rate (85·0%) than other regions did, *p* = 0·013). Continuous positive airway pressure (CPAP) was more frequently used as an initial (ranking score: 5·07 points) and postextubation (ranking score: 4·49 points) support modality than other NIV modes were (Fig. S1C-D).

#### Mechanical ventilation

Among the different MV modes, synchronized intermittent mandatory ventilation combined with pressure support ventilation and volume guarantee (SIMV + PSV + VG), synchronized intermittent mandatory ventilation combined with pressure support ventilation (SIMV + PSV), and pressure-controlled assist-control combined with volume guarantee (PC-AC + VG) were the top three modes used (ranking scores: 4·47, 4·09, and 3·92 points, respectively) (Fig. S1E). High-frequency oscillation ventilation (HFOV) was employed as a rescue therapy after conventional MV failure by 91·7% of the physicians, whereas only 36·9% chose it as the initial support modality for EPIs (Fig. S1F).

#### Caffeine therapy

A total of 59·1% (233/394) of the respondents reported routinely using caffein in patients receiving MV, and 29·2% (115/394) of them preferred to initiate caffeine therapy before weaning patients from ventilators. A total of 82·5% (325/394) of the doctors chose to start caffeine treatment as early as possible for preterm babies whose gestational age was less than the median gestational age of 32 weeks and whose birth weight was less than 1500 g.

#### Bedside lung ultrasound

Bedside lung ultrasound (LUS) was performed in 54·3% (214/394) of the hospitals (Table 4). Tertiary hospitals had great access to bedside LUS than secondary hospitals did (61·6% vs. 30·4%, *p* < 0·001). The primary applications of LUS are shown in Fig. S2. In the 180 hospitals without bedside LUS, lower-tier cities faced ultrasound machine shortages (1st-tier 36·2% vs. 2nd-tier cities 45·0% vs. 3rd-tier and lower-tier cities 61·8%, *p* = 0·006).

### Ancillary facility construction

Only 30·2% (119/394) of the hospitals possessed a qualified breast milk bank equipped to perform testing, sterilization, storage, and distribution. There was a greater proportion of tertiary hospitals than secondary hospitals (33·4% vs. 19·6%, *p* = 0·011), and there were fewer general hospitals than paediatric specialty hospitals (26·1% vs. 36·6%, *p* = 0·028). Home-like wards were available in only 36·0% (142/394) of the hospitals, with more paediatric specialty hospitals than general hospitals having these wards (48·4% vs. 28·2%, *p* < 0·001). Family Integrated Care (FICare) was available in 63·7% of the hospitals. A greater number of tertiary hospitals and paediatric specialty hospitals implemented FICare in their daily care (tertiary hospitals: 67·9% vs. secondary hospitals: 50·0%, *p* = 0·002). Among all these regions, central China had the highest proportion of these hospitals (81·8%) (Table S4). The comparison data are shown in Table 4.

### Medical insurance

**Fig. 3:**
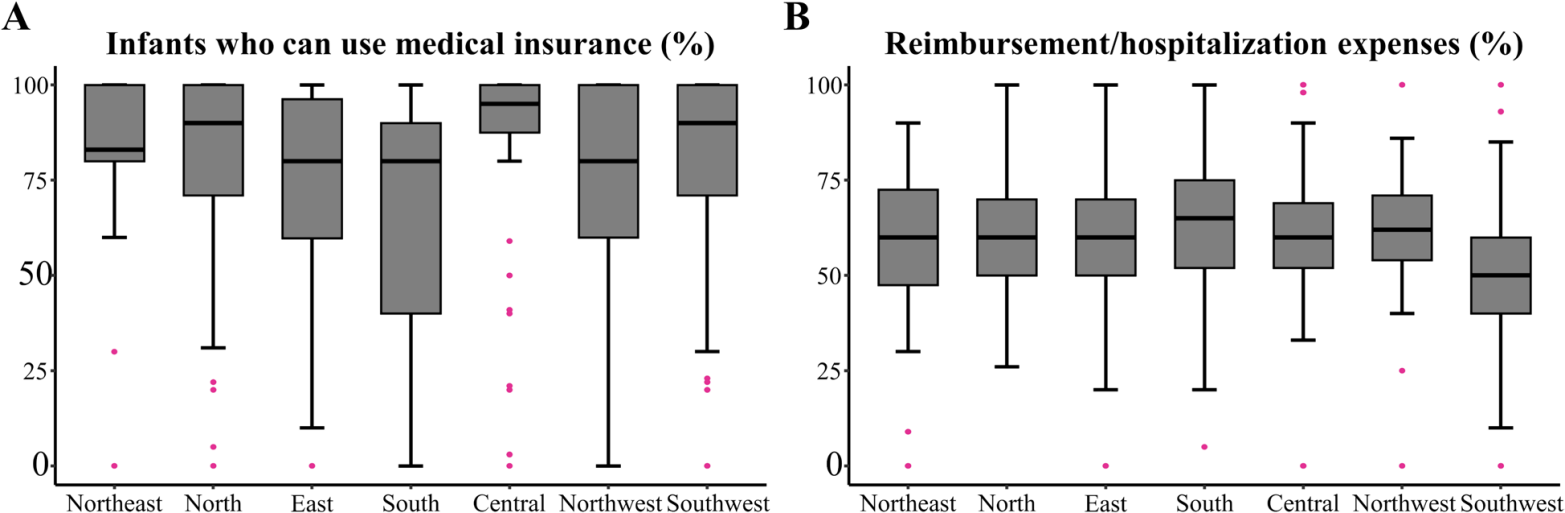
Variations in medical insurance among the different geographic regions (*p* < 0·05). **a** Infants with medical insurance, **b** Insurance reimbursement/hospitalization expenses

Approximately 90% of RDS patients in the hospitals were eligible for medical insurance reimbursement. The highest percentage (95%) was observed in Central China (Fig. 3A). The percentage of medical insurance reimbursement expenses concerning overall hospitalization expenses was approximately 60%, with Southern China having the highest proportion at 65% (Fig. 3B).

## Discussion

The MUNICH is a cross-sectional, nationwide online survey that aimed to provide a comprehensive overview of the current landscape of RDS care by collecting data from 394 neonatologists (hospitals) across China. The survey explored numerous facets of RDS management and concluded that neonatologists in China are well equipped with essential expertise for the effective treatment of RDS patients. As a result, several areas needing further improvement were identified, including low numbers of doctors and nurses per bed, a lower antenatal corticosteroid utilization rate among outborn infants, relatively conservative oxygen therapy use in DRs, less use of the LISA method, insufficient infrastructure support, and considerable inconsistencies in RDS care among hospitals. To our knowledge, this is the first national survey considering various aspects of RDS treatment in China.

In the survey, we observed median doctors per bed and nurses per bed ratios of 0·27 and 0·72, respectively. These figures closely align with previously published data (doctors per bed, 0.26; nurses per bed, 0.70) [19]. Disparities among different types of hospitals pose additional challenges. Paediatric specialty hospitals, which had more patients, experienced lower staffing ratios for both doctors and nurses. This imbalance may result in increased workload, potentially leading to lower job satisfaction and staff turnover, creating a detrimental cycle. A recent review revealed that the ratio of nurses per bed in neonatology departments in LMICs varies significantly, indicating an insufficient and inequitable distribution of health workers and a heavy workload in LMICs [20]. Presently, there are no globally accepted recommendations for staffing ratios in neonatology departments. However, UK standards by Bliss suggest that there should be a minimum nurse-to-baby ratio of 1:1 for intensive care [21]. A 1:1 NICU nursing staffing ratio has been associated with reduced in-hospital mortality, whereas understaffing increases the risk of nosocomial infections in very-low-birth-weight babies [21, 22]. Improving staff allocation and reducing imbalances may be pivotal steps in China.

Approximately 90·0% of preterm infants born at < 34 weeks of gestation received antenatal corticosteroids (any dose). The CHNN reported antenatal corticosteroid use in 75·6% of infants born at < 32 weeks of gestation [4]. In the US, 88·1% of mothers with extremely preterm babies receive antenatal corticosteroids [23]. Notably, there has been increasing emphasis on antenatal corticosteroid use over time [24–26]. However, our data highlight a potential concern: a lower antenatal corticosteroid utilization rate among outborn infants, with emergency labour being a primary factor. Therefore, mothers at high risk of preterm birth require systematic pregnancy management and should be transferred to experienced perinatal centres.

Our data revealed that 82·0% of the DRs in the hospitals had an oxygen blender, which was slightly lower than the 91% reported in Europe [27]. The initial FiO_2_ setting was 0·30 for babies born at ≤ 32 weeks of gestation and 0·25 for those born at > 32 weeks of gestation, which appeared more conservative than what has been recommended in guidelines and consensuses [7, 8]. Notably, general hospitals tended to have higher initial FiO_2_ settings than paediatric specialty hospitals did. Regarding target oxygen saturation after the first 10 min postnatally, tertiary hospitals tended to be more consistent with the guidelines and consensuses than secondary hospitals did (90·0%-95·0% vs. 88·0%-95·0%, *p* < 0·05). The overall availability rate of TPRs in the DRs was 77·8%. Disparities were identified among hospitals and across regions. Despite the common perception of greater development in East and South China, less developed Northwest China exhibited better leadership in this respect. This could be attributed to the efforts and emphasis placed on the regional neonatal network and neonatal societies in Northwest China. Moreover, only 12% of infants born at < 32 weeks of gestation received CPAP in the DR according to CHNN data, which is significantly lower than 79% reported in European data [27]. This highlights a substantial gap between possession and utilization, underscoring the urgent need for future TPR promotion and training [4]. Of the 48 respondents who had only bag-valve-mask resuscitators in the DR, the first step to improvement is to have the right equipment in place.

There are certain inconsistencies concerning where, when, and how to administer PS. Surfactant was not available in the DRs in 48·4% of the birthing centres, and maternity-child healthcare hospitals had greater access to surfactant than general hospitals did. The reasons cited included the inability to obtain surfactant from the hospital pharmacy, lack of reimbursement before birth, and potential refusal by parents to cover the cost of PS. These issues underscore the challenges of medication access in DRs and prenatal communication, necessitating multidisciplinary collaboration for resolution. Doctors have displayed a variety of approaches in which FiO_2_ is used as an indicator for PS administration in patients receiving NIV, despite many studies suggesting that a FiO_2_ of 0·30 predicts CPAP failure and the need for PS [28–32]. According to our data, 49·7% of the hospitals performed LISA. A previous survey indicated that the LISA adoption rate was 52% in Europe, and a recent survey in Turkey reported that it was 81·6%, emphasizing the growing prevalence of LISA/MIST techniques in recent years [33, 34]. In China, the LISA/MIST utilization rate is relatively low, and more training is needed.

The survey revealed significant variations and imbalances among different hospitals, with tertiary hospitals and paediatric specialty hospitals having greater access to advanced medical resources. Less developed regions face more technical barriers, and medical insurance policies vary across different districts. This situation is a result of both socioeconomic and medical factors. Drawing upon the data from the MUNICH, the following inferences can be made: (1) NICU admission criteria for hospitals of various levels and types and centralized management of extremely and very preterm infants or newborns requiring advanced life support are needed. Ideally, in utero transfers can be performed for high-risk pregnant mothers. (2) Training sessions such as DR resuscitation strategies, LISA/MIST techniques, and ventilation strategies are needed. (3) The promotion of ancillary facilities, including qualified breast milk banks, home-like wards, and FICare is needed to enhance medical care in key hospitals. (4) Improvements in medical insurance policies and reimbursement rates are requested to provide solid support for the treatment of preterm infants. Next, we need to work with the Subspecialty Group of Neonatology and CHNN to widely disseminate the latest RDS guidelines and consensuses, and a future survey with a larger sample size is advisable. Today, the paediatricians’ responsibility is not only to heal, but also to promote the child’s well-being (mental, physical and social) [35]. Through these efforts, it is hoped that neonatologists will be better able to assist families in making the right decisions from a medical, social, political and economic perspective.

This survey has several limitations. First, collecting data online does not ensure the accuracy of the numerical values obtained. Second, discrepancies may arise between the perspectives of physicians and the actual information. Third, the sample size of paediatric specialty hospitals included in this survey was relatively limited, which may restrict the generalizability of the findings to the broader population of neonatal RDS patients. Notably, the sample distribution was not fully aligned with what was planned due to response variations. Moving forward, our next step involves collaborating closely with the Subspecialty Group of Neonatology and CHNN to ensure broad dissemination of the updated RDS guidelines and consensuses. Additionally, an expanded survey with a larger cohort is expected to enrich our insights.

## Conclusions

Chinese medical practitioners possess the necessary expertise to address the diverse requirements associated with RDS care effectively. However, certain deficiencies and significant variations exist. Enhancing staff allocation, upgrading DR facilities and medications, overcoming gaps in key techniques, fostering multidisciplinary collaboration, and developing ancillary facilities will contribute to the overall improvement in RDS management.

## Supplementary Information

Supplementary Material 1.

## Data Availability

The datasets generated during and/or analyzed during the current survey are available from the corresponding author upon reasonable request.

## Abbreviations

CHNN: Chinese Neonatal Network
CI: Confidence interval
CPAP: Continuous positive airway pressure
DR: Delivery room
EPI: Extremely preterm infant
FICare: Family integrated care
FiO_2_: Fraction of inspired oxygen
HFOV: High-frequency oscillation ventilation
INSURE: INtubation-SURfactant-Extubation
LISA: Less invasive surfactant administration
LMICs: Low- and middle-income countries
LUS: Lung ultrasound
MIST: Minimally invasive surfactant therapy
MUNICH: Medical sUrvey of NICU Insight in China
MV: Mechanical ventilation
NIV: Non-invasive ventilation
NICU: Neonatal intensive care unit
PC-AC: Pressure-controlled assist-control
PS: Pulmonary surfactant
PSV: Pressure support ventilation
RDS: Respiratory distress syndrome
SIMV: Synchronized intermittent mandatory ventilation
TPR: T-piece resuscitator
VG: Volume guarantee
